# Beyond visualizing the bird beak: esophagram, timed barium esophagram and manometry in achalasia and its 3 subtypes

**DOI:** 10.1007/s00261-024-04554-8

**Published:** 2024-09-24

**Authors:** Lindsay Duy, Steven Clayton, Nayeli Morimoto, Shery Wang, David DiSantis

**Affiliations:** 1 Department of Radiology, Wake Forest University School of Medicine, Winston Salem, USA; 2 Department of Gastroenterology, Wake Forest University School of Medicine, Winston-Salem, USA; 3 Department of Radiology, Stanford University School of Medicine, Stanford, USA; 4 Department of Radiology, Mayo Clinic, Rochester, USA; 5 Department of Radiology, Mayo Clinic, Jacksonville, USA

**Keywords:** Achalasia, Timed barium esophagram, Esophagram, Manometry

## Abstract

Achalasia is a rare esophageal motility disorder characterized by lack of primary peristalsis and a poorly relaxing lower esophageal sphincter. This disease process can be examined several ways and these evaluations can offer complementary information. There are three manometric subtypes of achalasia, with differing appearances on esophagram. Differentiating them is clinically important, because treatment for the subtypes varies. Timed barium esophagram (TBE) is a simple test to quantitatively evaluate esophageal emptying. TBE can be used to diagnose achalasia and assess treatment response. Considerable variation in the TBE protocol exist in the literature. We propose a standardized approach for TBE to allow for comparison across institutions.

## Introduction

Achalasia is the classic example of an esophageal motility disorder. This was first described in 1674 by Sir Thomas Willis in a patient with chronic dysphagia requiring dilation of the lower esophageal sphincter (LES) with a sponge attached to a carved whalebone [1].

**Fig. 1:**
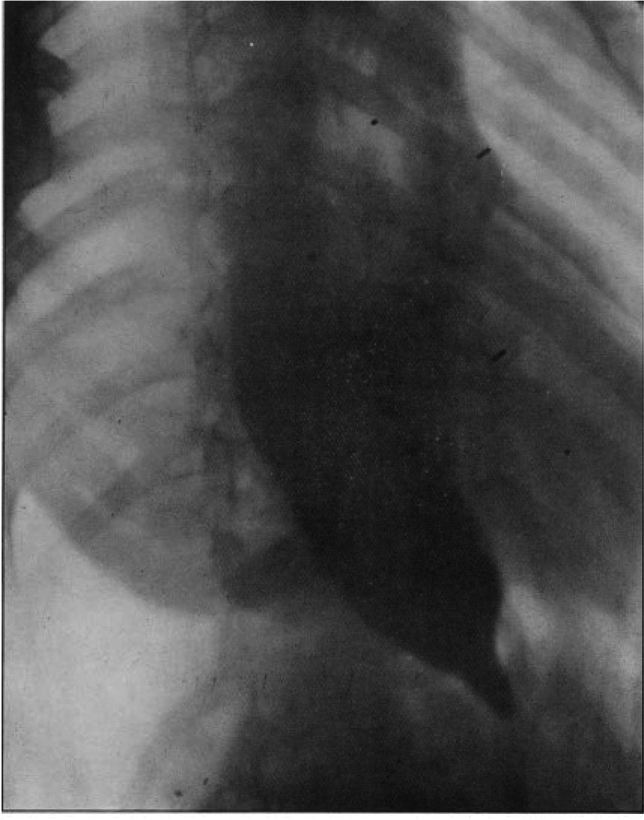
From Hertz, A.F., *Achalasia of the Cardia* (*so-called Cardio-spasm*)*.* Proc R Soc Med, 1915. **8**(Clin Sect): p. 22–25. Used with permission

The modern medical term achalasia first appeared in the medical literature in an article by Arthur Hertz in 1915. This article describes one of the earliest radiographic evaluations of the esophagus in a patient with achalasia. This “skiagram” (esophagram) was performed by Dr. Linsay Locke. During this evaluation, the patient was given a bismuth meal. The early esophagram was interpreted as “the whole oesophagus was dilated, the obstruction being at the cardiac orifice of the stomach. There was violent peristalsis in the oesophagus, but the food only trickled with extreme slowness- into the stomach” (Fig. 1).

Based on these radiographic findings, Dr. Hertz and his colleague, Sir Cooper Perry, coined the term “achalasia” (in Greek: a, not; χαλάω, I relax) to describe the failure of the LES to deglutitively relax resulting in a functional obstruction to bolus passage at the esophagogastric junction, EG-J [2, 3]. In addition, there is also loss of the esophageal peristaltic function. The combination of a functional obstruction at the LES and esophageal aperistalsis results in esophageal bolus stasis leading to symptoms of dysphagia and voluminous bland regurgitation [4, 5].

Achalasia is a rare disorder with an annual incidence of 1–3.1 per 100,000 people and a prevalence of 10–45 per 100,000. The exact cause for achalasia is unclear. Viral infection, autoimmune response, and genetic susceptibility factors have been implicated as possible causes or contributing etiologies for the neurodegenerative loss of ganglion cells in the myenteric plexus of the esophagus and LES [6]. It is a chronic disease with a very low mortality rate and typically presents between the third and sixth decades of life but can occur in any race and at any age, or gender [1]. Achalasia does occur in the pediatric population with an estimated annual incidence of 0.11% in children less than age 16.5 [7]. In 2021, a study of two large administrative claims databases in the United States concluded that achalasia imposed a higher epidemiologic and economic burden than previously suggested, with a total direct medical cost exceeding $408 million in 2018 [8]. The purpose of this paper is to describe the demographics, diagnosis, and radiographic features of achalasia and its three subtypes. A secondary aim is to suggest a standardized method of performance and interpretation of the timed barium esophagram.

## Diagnosis

### Endoscopy

Endoscopy, barium esophagram, and esophageal manometry are useful in making the diagnosis of achalasia in patients with clinical symptoms concerning for achalasia. Endoscopic findings of retained saliva or food in the esophagus, a dilated esophageal lumen and a puckered appearance at the EG-J support the diagnosis of achalasia. Endoscopy is required to exclude malignancy or other forms of mechanical obstruction at the EG-J which can mimic symptoms of achalasia (pseudoachalasia) [9, 10].

### Manometry

High-resolution manometry (HRM) is the gold standard for diagnosing disorders of esophageal motility, including achalasia [11]. The HRM catheters consist of solid-state pressure sensors spaced 1 cm apart. The catheter is positioned in the esophagus to span the length extending from the hypopharynx to the stomach. Computer software generates esophageal pressure topography (EPT) plots that represent esophageal motility and sphincter function on color-coded, pressure-space–time plots called a Clause plot [12]. We will now briefly review HRM metric terminology and clinical significance. The key metrics used to describe esophageal motility on HRM analysis are the integrated relaxation pressure (IRP), the distal contractile integral (DCI), the distal latency (DL) and pressurization.

Achalasia is considered a disorder of esophagogastric outflow obstruction and defined as having an elevated median IRP (> 15 mmHg) without preserved esophageal smooth muscle peristalsis [11, 12]. The IRP is of paramount importance in the diagnosis of achalasia. The IRP is the lowest nadir pressure of the LES that occurs during a swallow [12]. Normative IRP values vary depending on the manufacturer of the manometry equipment but a median value for 10 supine swallows of less than 15 mmHg is considered normal. As the poor deglutitive relaxation of the LES is the key feature of achalasia an elevated median IRP is concerning for the diagnosis.

The DCI measures the peristaltic contractile vigor and contractile pattern. Normal values for DCI are between 450 and 8000 mmHg s cm. A DCI less than 450 mmHg s cm is suggestive of esophageal hypomotility and greater than 8000 mmHg s cm is suggestive of a hypercontractile peristalsis. A DCI less than 100 mmHg s cm is consistent with absent or failed peristalsis. Failed peristalsis with an elevated median IRP is suggestive of type I achalasia [11].

DL is measured as the interval from the upper esophageal sphincter relaxation to the contractile deceleration point (CDP). The CDP is the inflexion point where propagation velocity slows, demarcating bolus transit from the tubular esophagus to the phrenic ampulla. A swallow is defined as a premature contraction when the distal latency is shorter than 4.5 s, in the setting of a DCI of 450 mmHg s cm or greater. A shortened DL is suggestive of an uncoordinated or spastic swallow. A shortened DL in conjunction with an elevated median IRP is suggestive of type III achalasia [11].

**Fig. 2:**
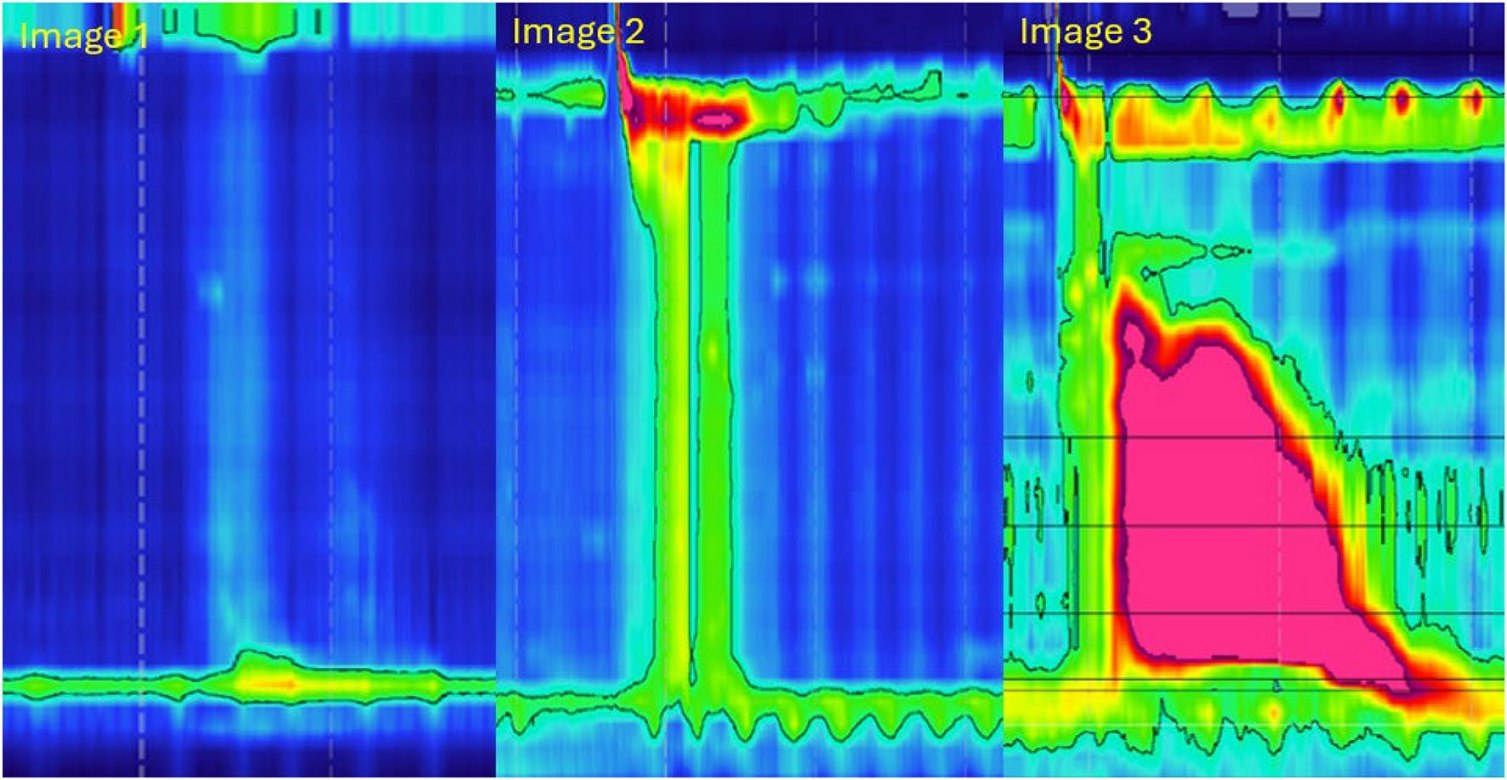
Legend—X-axis is time in seconds, Y-axis is distance in centimeters. In high-resolution manometry, colors are used to represent pressure in a color contour plot. The cooler colors (blue and green) represent lower pressures and warmer colors (red and orange) representing higher pressures. High resolution manometry allows achalasia to be differentiated into three subtypes based on manometric patterns. X-axis is time in seconds, Y-axis is distance in centimeters. In high-resolution manometry, colors are used to represent pressure in a color contour plot. The cooler colors (blue and green) represent lower pressures and warmer colors (red and orange) representing higher pressures. Image **1** Type I achalasia (classic) has absent smooth muscle contractility in the esophageal body with an elevated median IRP > 15 mmHg. Image **2** Type II achalasia has ≥ 20% of swallows of panesophageal pressurization and an elevated median IRP > 15 mmHg. Image **3** Type III achalasia (spastic/vigorous) has shortened distal latency (< 4.5 s), DCI > 450 mmHg cm s and an elevated median IRP > 15 mmHg

Finally, subtyping of achalasia first began in 2008 with Chicago classification scheme after the advent and adoption of the high resolution manometry catheter [13]. In defining the subtypes of achalasia, there is the concept of panesophageal pressurization. Panesophageal pressurization is the luminal pressure exerted on the whole esophageal body and abnormal is defined as being greater than 30 mmHg using the isobaric contour tool. Panesophageal pressurization is indicative of esophageal obstruction to bolus transit. Panesophageal pressurization with an elevated median IRP is suggestive of type II achalasia [11]. The formal manometry diagnostic criteria for the subtypes of achalasia are as follows:

Type I (classic achalasia) has absent contractility in the esophageal body with an elevated median IRP, type II has ≥ 20% of swallows of panesophageal pressurization and an elevated median IRP, and type III (spastic/vigorous) has shortened distal latency (< 4.5 s), DCI > 450 mmHg cm s and an elevated IRP. Figure 2 shows representative swallow examples of the three subtypes of achalasia. Subtyping achalasia is of clinical importance as the three subtypes clinically present similarly, but treatment response varies considerably between the three subtypes [3, 11, 14, 15].

### Barium esophagram

A barium esophagram can reveal the findings of achalasia and assess for other causes of dysphagia [1]. Symptoms of achalasia, for example, regurgitation, can overlap with those of GERD [5]. Double contrast technique can help detect mucosal abnormalities which can be seen in pseudoachalasia. Double contrast is achieved by asking the patient to swallow effervescent crystals with a small amount of water, followed by swallowing a few sips of high-density barium [16, 17]. Upright images should be obtained in the left posterior oblique (LPO) position to position the esophagus off the vertebral bodies. This can show smooth tapering or “bird’s beak” at the lower esophageal sphincter (LES), a standing column of contrast, and esophageal dilation [3, 18–21]. Esophageal motility should be assessed in the prone right anterior oblique (RAO) position. This removes the assistance of gravity in esophageal emptying. The patient should be instructed to swallow a single sip of barium to observe a single wave of peristalsis [16, 21]. Asking the patient to open their mouth after swallowing keeps the patient from swallowing a second time. This swallow should be observed and stored as a video clip and is repeated 3–5 times. Motility is considered abnormal when abnormal peristalsis is seen on at least 2 swallows out of 5 [21, 22].

**Fig. 3:**
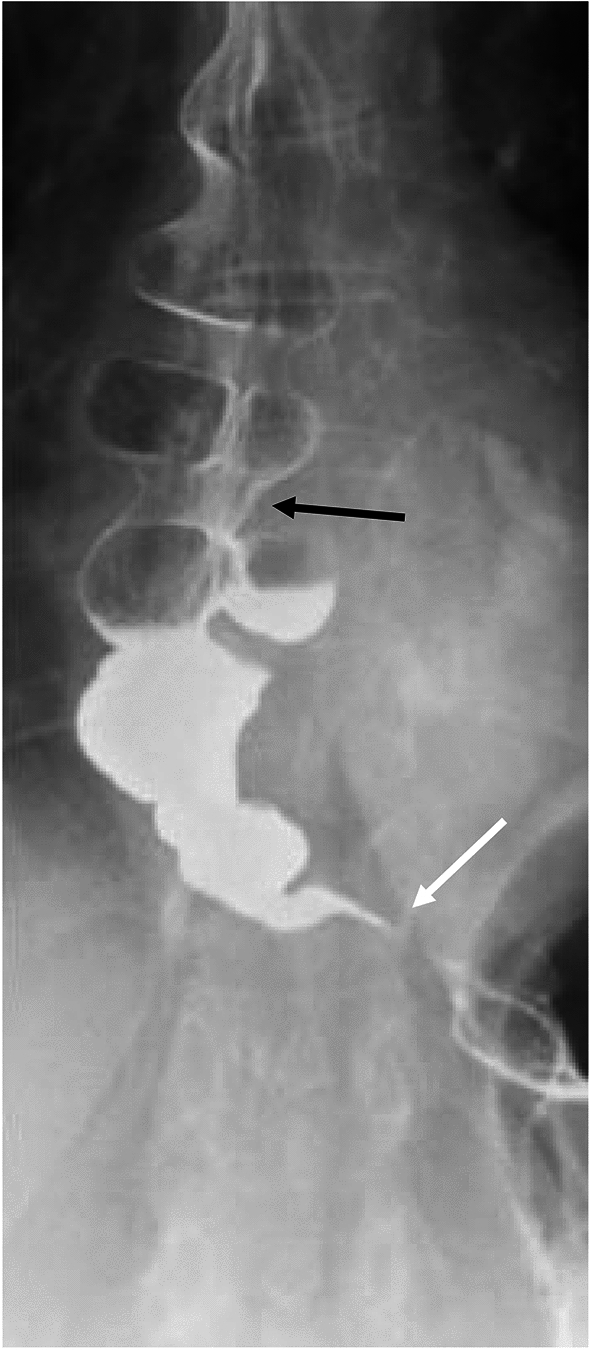
Fluoroscopic image of type III achalasia shows multiple segmental contractions (black arrow) with smooth tapering at the LES (white arrow)

**Fig. 4:**
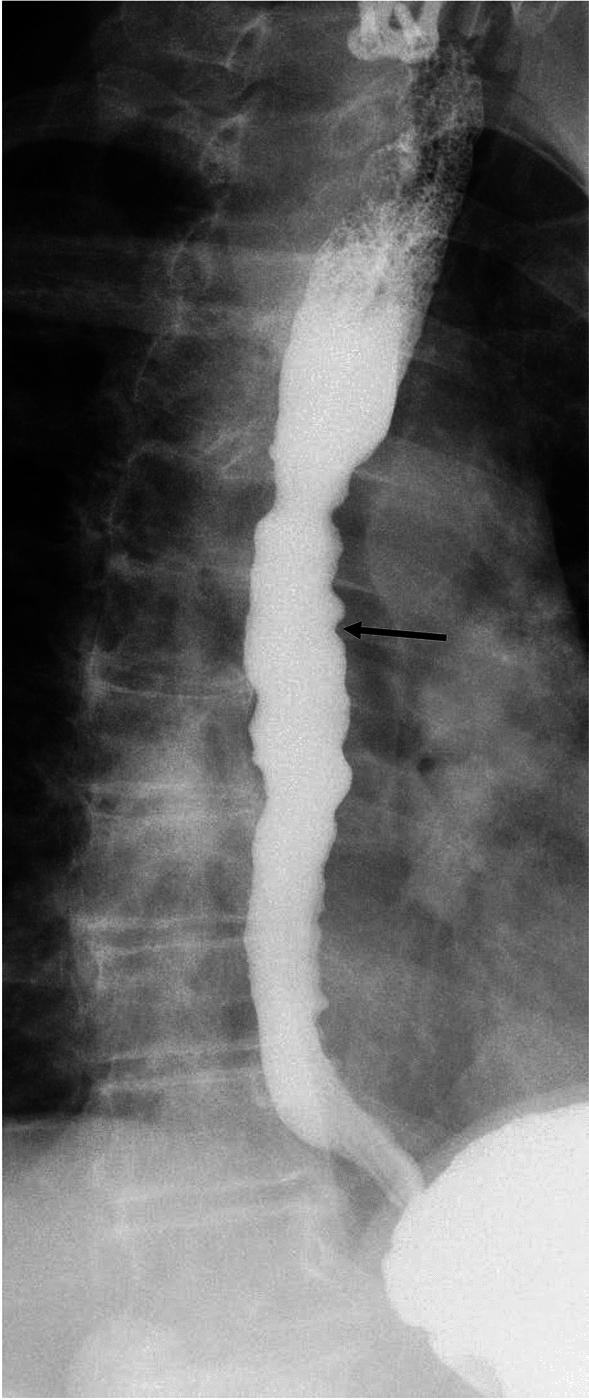
Fluoroscopic image showing type II achalasia. The esophagus is nondilated, and there are contractions that indent but do not obliterate the esophageal lumen (arrow)

**Fig. 5:**
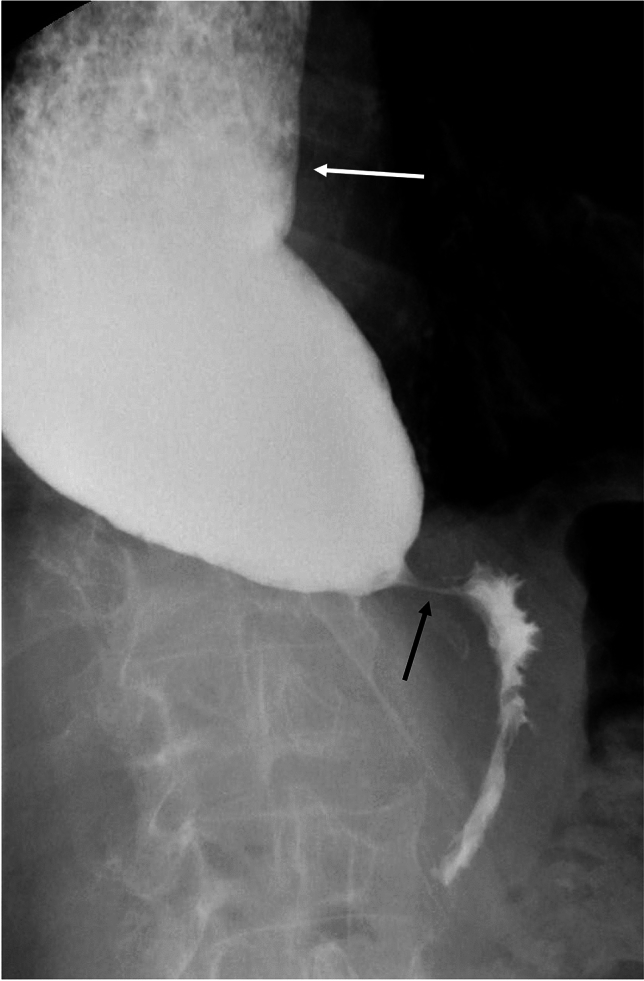
Fluoroscopic image showing type I achalasia. Esophagus is dilated, with fluid level on this upright image (white arrow). There is smooth tapering at the LES (black arrow)

There are key findings on barium esophagram common to all types of achalasia. Absence of primary peristalsis and incomplete relaxation and smooth tapering of the distal most esophagus are common to all types [21]. Additionally, liquid barium stasis in the upright position strongly supports the diagnosis of achalasia [23]. There are differences between the subtypes of achalasia that can be seen on manometry and esophagram (Table 1). Some experts hypothesize that achalasia progresses from type III to type I, correlating with progressive ganglion cell loss [24]. Type III achalasia is historically known as spastic or vigorous achalasia [5, 11]. In type III achalasia, there are spastic, circumferential contractions which obliterate the lumen (Fig. 3) referred to as the corkscrew sign or rosary bead sign [25, 26]. The esophagus is usually non-dilated in type III achalasia [18, 27]. Type II achalasia can be difficult to diagnose on the basis of esophagram alone. The appearance is variable and esophageal dilation can be seen, but is not as frequently as with type I [18]. Nonpropulsatile contractions that indent but not obliterate the lumen may be present (Fig. 4). In type I achalasia the esophagus is usually dilated, with minimal or no contractions (Fig. 5) [18, 28]. Type I achalasia is historically referred to as classic achalasia [11].

**Table 1:** Discriminatory findings between types of achalasia on manometry and esophagram

Achalasia type		I	II	III
Manometry		Absent contractility	Panpressurization	Shortened distal latency
Standard Esophagram	Contractions	Minimal or no contractions	Nonpropulsatile contractions that do not obliderate the lumen	Spastic tertiary contractions
	Esophageal Caliber	Dilated	Variable	Normal

**Fig. 6:**
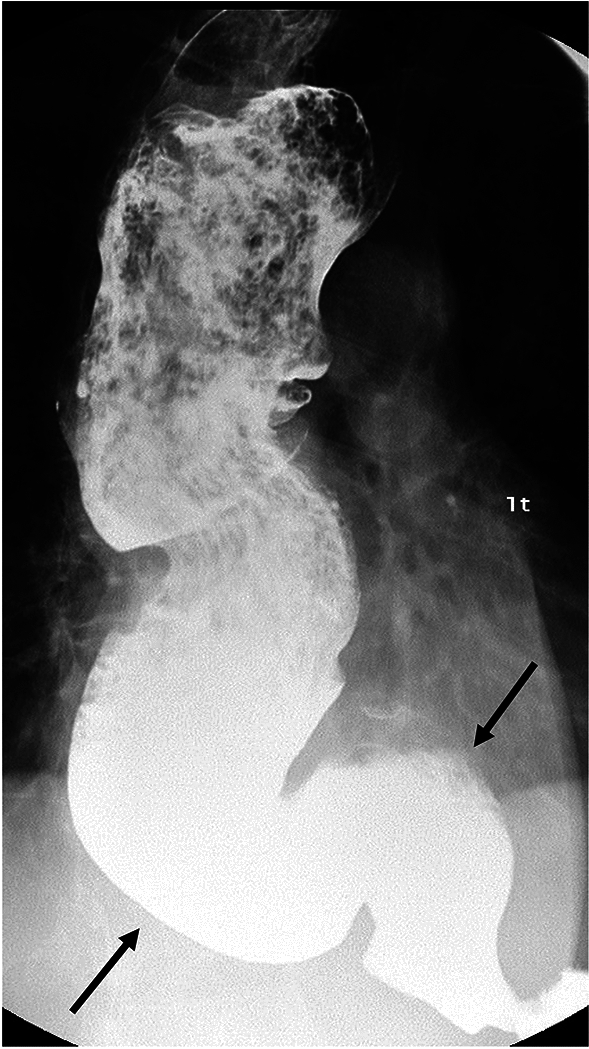
Fluoroscopic image showing sigmoid esophagus. The esophagus is markedly dilated and tortuous, with angulation of the esophagus (arrows)

As esophageal dilation progresses, the esophagus becomes tortuous which is known as a sigmoid esophagus (Fig. 6). The degree of esophageal dilation and esophageal angulation is important to report. These patients have poorer outcomes with pneumatic dilation, surgical myotomy, or peroral endoscopic myotomy (POEM) as the angulation of the esophagus impedes gravity-assisted emptying. Consequently, they may require enteral feeding or esophagectomy [3, 29, 30].

**Fig. 7:**
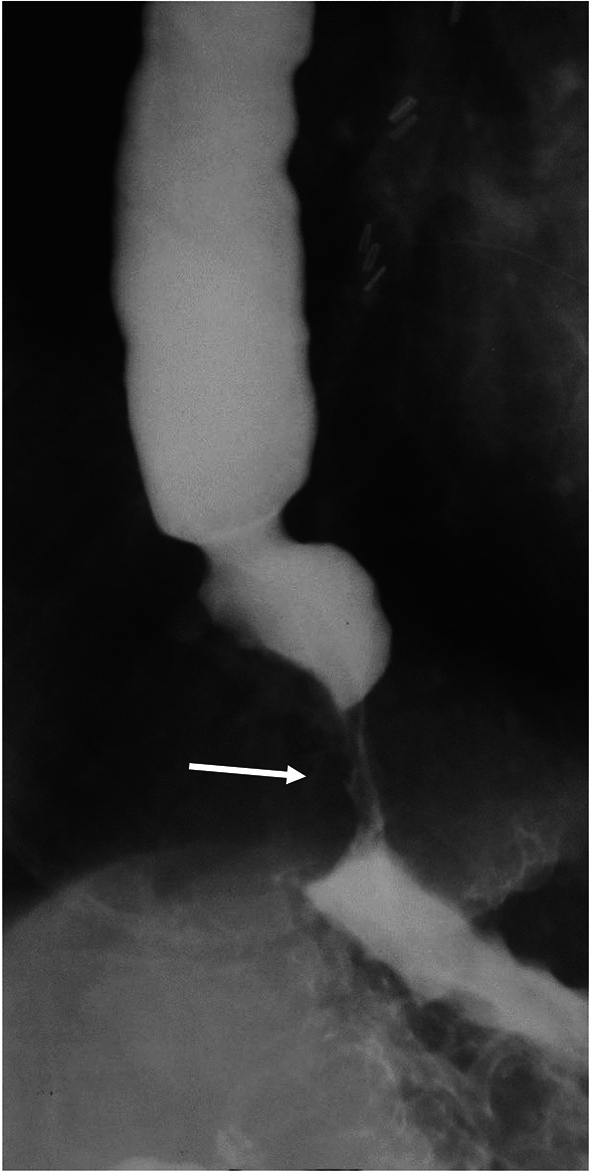
Fluoroscopic image of distal esophageal carcinoma. There is longer segment narrowing of the distal esophagus than is typical of achalasia, and the narrowed segment is neither smooth nor centric (arrow)

Barium esophagram can also suggest the diagnosis of pseudoachalasia (secondary achalasia), or tumor at the EG-J (Fig. 7). This causes narrowing at the LES and can secondarily cause abnormalities in motility. Nodularity, irregularity, and ulcerations can be seen at the site of tumor [21]. Secondary achalasia is also associated with eccentric, longer segment narrowing at the EG-J than primary achalasia [17, 21].

## Timed barium esophagram

The timed barium esophagram (TBE) was initially described as a simple radiographic examination to evaluate esophageal emptying in patients with achalasia and the intent was to define and simplify a noninvasive, reproducible way to assess esophageal emptying in an objective manner. The patient would swallow 100 to 200 mL low-density barium sulfate (45% weight in volume), as best able to tolerate without regurgitation or aspiration. Radiographs were obtained in the LPO upright position at 1 min, 2 min, and 5 min after ingestion. Other than pixel counts, quantitative esophageal emptying was also determined by measuring the height and width of the column at 1 and 5 min to calculate a simple area and percent emptying before and 1 month after treatment with either pneumatic dilatation or *Clostridium botulinum* injection [31]. Over time, this technique has been modified across numerous studies, the emphasis on the types and importance of quantitative measurements has changed, and its utility in assessing achalasia tested as the understanding of the disease process of achalasia and its various interventions have evolved.

### Modifications of the technique

Despite modification of the technique over time, low-density barium sulfate remains the contrast medium of choice. Ingested volumes are variable, ranging from 100 to 250 mL [16, 27, 31–39]. The time over which the barium was ingested ranged from 15 to 45 s [16, 27, 31, 33, 39]. Upright LPO radiographs continue to be a consistent methodology for the radiographic evaluation of esophageal emptying. Although many studies describe obtaining radiographs at 1 min, 2 min, and 5 min, the 5-min radiograph is not necessarily needed if contrast is cleared by the 2-min radiograph [31]. Ideally, pre- and post-treatment TBEs should be performed on patients suspected of having achalasia but follow-up was variable among studies, ranging from 1 month post-treatment [31, 32, 38], 3 to 6 months post-treatment [33] to 10 years post-treatment [34]. More recently, there has been advocacy for lifelong follow-up [40]. The addition of a 13 mm barium tablet is another modification to the originally described technique [35].

### Quantitative measurements

The large procedural variability of the timed barium esophagram performed adds to the complexity of interpretation due to lack of standardization of its technique.

**Fig. 8:**
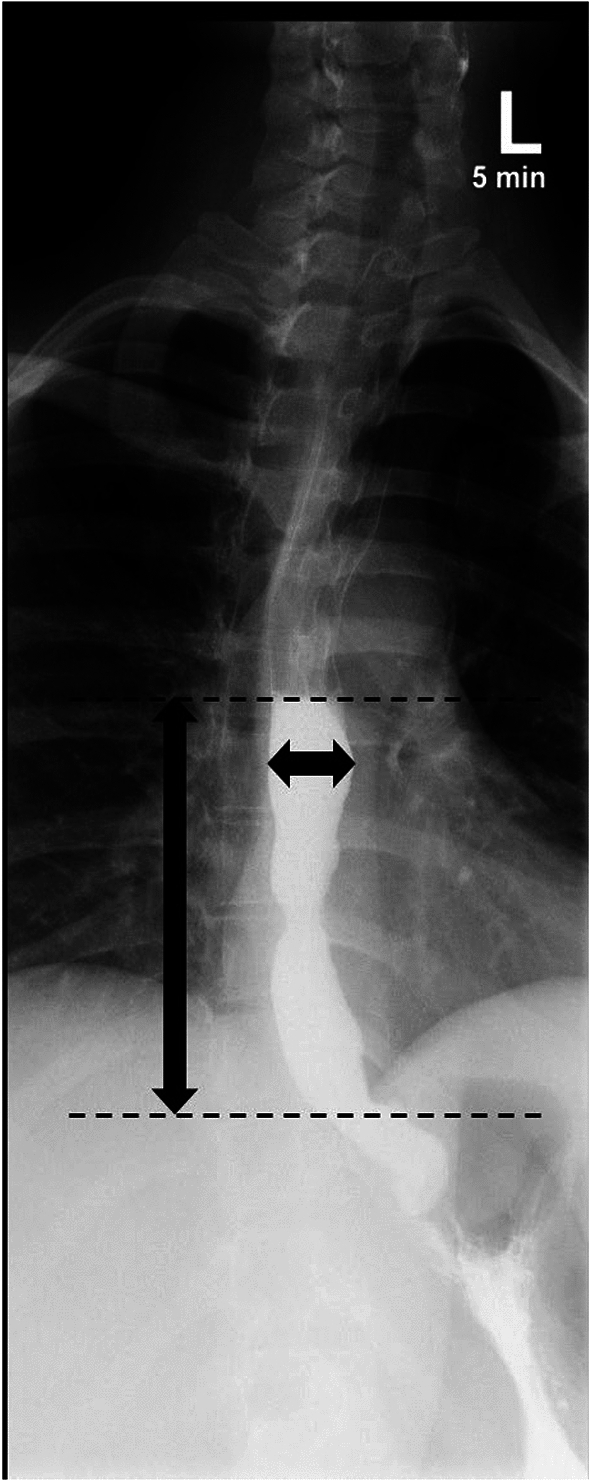
Following ingestion of barium, an LPO radiograph at 5 min demonstrates a columnation of contrast. Vertical arrow shows column height. Horizontal arrow shows column width

Studies have examined column height, width, surface area, percent clearance, volume, and percent change in column height [27, 32, 33, 37, 38, 40, 41]. We suggest measuring both column height and width. Several studies focused on the column height as having predictive value for diagnosis of and/or determination of effective treatment of achalasia [27, 32, 34–36, 38, 40, 42, 43]. It is important to note that the height of the column should be measured vertically from the EG-J to the foam–barium interface, defined as the length of the standing barium column from the interface of the debris and barium of the column to the EG-J (Fig. 8). Width should also be reported because this has been shown to correlate with achalasia type and prognosis, further discussed in the interpretation and key findings subsection below [27, 32]. The width should be obtained as the maximum width of the column, perpendicular to the long axis of the column (Fig. 8) [39].

### Suggested technique

**Fig. 9:**
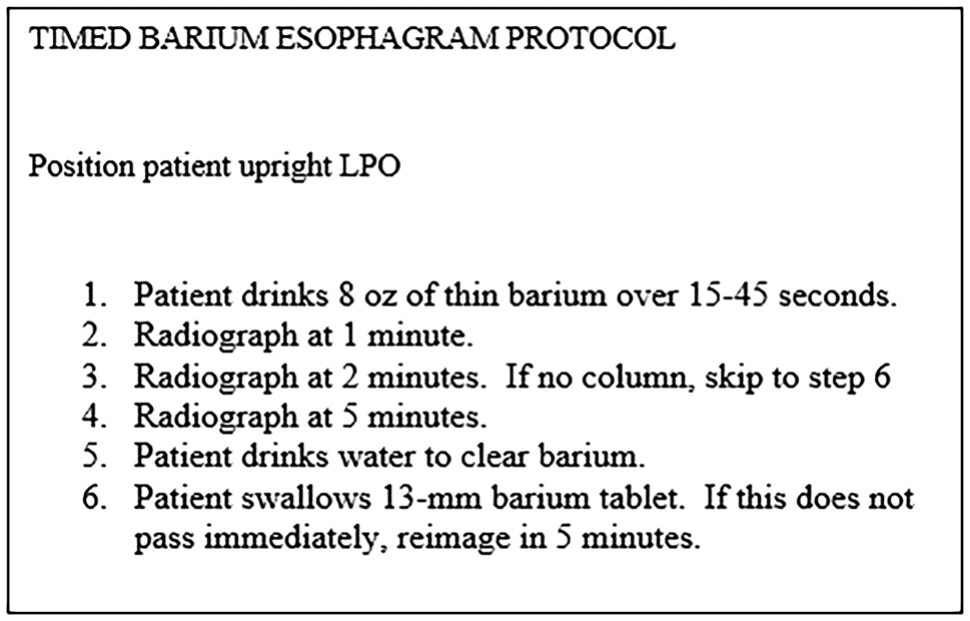
Proposed timed barium esophagram protocol

Blonski et al. [35] validated their protocol to diagnose achalasia based on column height at 5 min. To our knowledge, this is the only protocol that has been validated in this way. Therefore, we propose a standardized protocol based on their research. The patient should drink 8 oz of low-density barium sulfate (45% weight in volume) in an upright LPO position. If the patient cannot tolerate 8 oz without regurgitation or aspiration, the amount tolerated should be reported and subsequent follow-up exams should use the tolerated amount. The patient should attempt to drink the barium between 15 and 45 s [16, 27, 31, 33, 39]. Radiographs should be obtained in an upright LPO position at 1, 2, and 5 min. If there is no measurable column at a timepoint, the subsequent timepoints do not need to be obtained. If a column remains at 5 min, the patient should be given water to clear barium to allow visualization of a 13-mm barium tablet. A tablet should then be administered. If the tablet does not rapidly pass the EG-J, this should be given 5 min to pass (Fig. 9) [35].

### Interpretation and key findings

**Fig. 10:**
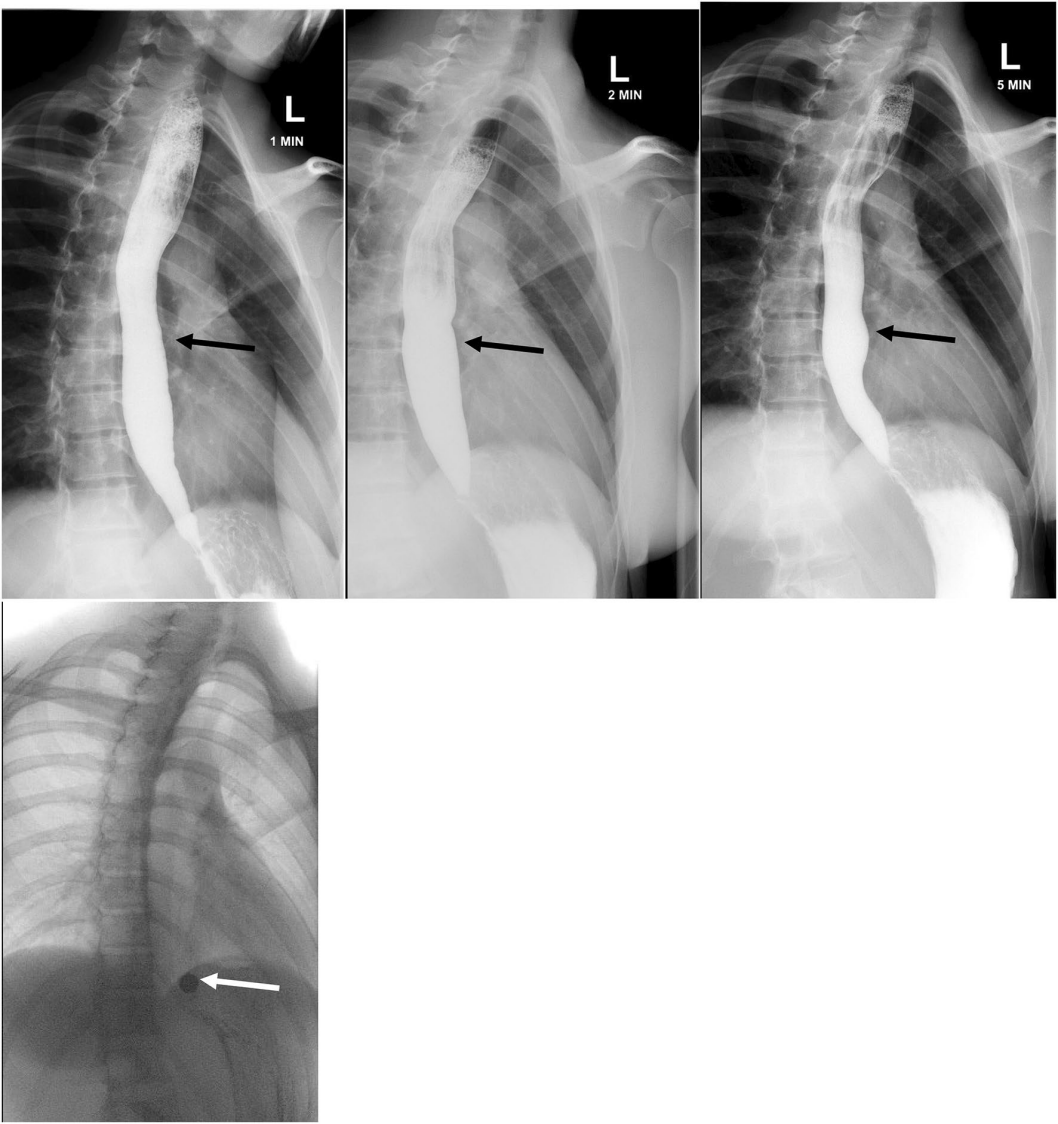
Abnormal TBE showing columns at 1, 2, and 5 min (black arrows). The barium tablet did not pass after 5 min (white arrow)

As the technique and data derived from the TBE have evolved, so has the understanding of its predictive value. In general, most healthy individuals empty their esophagus by 1 min and all healthy individuals empty by 5 min [32]. The barium tablet is a helpful addition to the TBE. Abnormal tablet retention at 5 min is indicative of untreated achalasia in combination with abnormal findings on the TBE, increasing diagnostic yield from 79.5 to 100% (Fig. 10) [35].

TBE is complimentary to manometry in achalasia patients. Starting with a volume of 8 oz (236 mL), barium height of > 5 cm after 1 min is highly sensitive (94%) for untreated achalasia. A barium height of > 2 cm after 5 min is both very sensitive (85%) and specific (86%) for untreated achalasia, as distinguished from non-achalasia conditions or EG-J outflow obstruction, EGJOO [35].

Earlier studies demonstrated that achalasia patients with > 50% improvement between pre- and post-treatment column height are unlikely to report significant and persistent dysphagia [38]. Additionally, percent change of 3% in column height pre- and post-treatment at 5 min is a better indicator of treatment success as compared with an absolute cutoff of 5 cm [41]. Most patients with reported improved symptoms also concordantly demonstrated improved esophageal emptying after treatment [32]. Height of column of the TBE at 5 min and the patient’s Eckhardt score significantly improved in patients with resolved achalasia pattern on high resolution manometry [43]. In patients with achalasia, poor emptying on TBE after treatment correlates well with future treatment failure despite symptomatic relief as evidenced in several studies. For example, approximately 30% of achalasia patients reporting complete symptomatic relief after pneumatic dilatation demonstrate poor esophageal emptying on initial post-procedural TBE. Of these patients, approximately 90% of these patients symptoms return within 1 year [38]. Similarly, post-procedure esophageal emptying of ≤ 40% indicates that moderate to severe achalasia will likely persist [33]. Additionally, the sensitivity to predict the need of retreatment is higher for esophageal stasis on TBE compared to LES pressure on manometry. Patients with poor emptying on post-treatment TBE have an 8-fold increased risk for retreatment compared with patients without stasis, irrespective of initial symptoms or LES pressure on manometry [34].

**Fig. 11:**
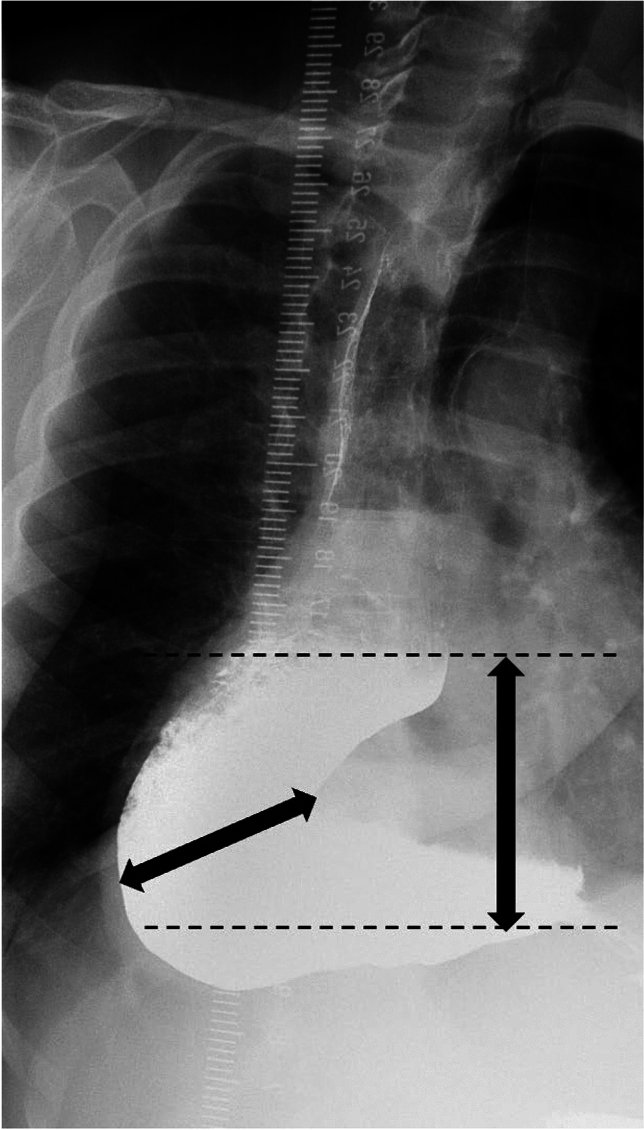
An example of column height and width measurements. LPO radiograph 5 min after ingestion of barium in type 1 achalasia in a patient with a sigmoid esophagus

TBE may also be helpful in differentiating achalasia types and prognosis. For example, a very dilated sigmoid configuration esophagus has a worse prognosis (Fig. 11) [43]. Additionally, achalasia patients with esophageal diameters > 8 cm do poorly after pneumatic dilatation and myotomy with fundoplication, likely because the esophagus remains baggy and dysfunctional with no tone [32], requiring more frequent retreatment [40]. Column height at 5 min was higher in achalasia types I and II than for III [27]. Although there is no relationship between maximal esophageal diameter on TBE and the Eckhardt score [43], column width may aid in stratifying types of achalasia and the need for retreatment. Column width is greater for type I, narrower in type II, and narrowest in type III and increasing width correlates with deteriorating morphology and function from type III to type I [27]. Change in column width of more than 3 cm at 1 min after myotomy is a risk factor for reintervention [40].

## Conclusion

In summary, achalasia is a rare motility disorder with a significant economic burden in the United States. Manometry is the gold standard for diagnosis and typing of achalasia, but barium esophagram can also be of value. Accurate diagnosis of achalasia type is important because treatment varies with type.

The TBE has evolved since its introduction and aids in the diagnosis and stratification of types of achalasia. This examination may also be useful as an alternative strategy for monitoring treatment response in patients with treated achalasia. The barium tablet also provides more information and can be added to the TBE protocol. Ultimately, a standard technique specifying the exact volume, timing, and data acquisition would be optimal to allow a standardized approach to interpretation across institutions.

## Data Availability

No datasets were generated or analysed during the current study.
